# Using Innovative Acoustic Analysis to Predict the Postoperative Outcomes of Unilateral Vocal Fold Paralysis

**DOI:** 10.1155/2016/7821415

**Published:** 2016-09-21

**Authors:** Yung-An Tsou, Yi-Wen Liu, Wen-Dien Chang, Wei-Chen Chen, Hsiang-Chun Ke, Wen-Yang Lin, Hsing-Rong Yang, Dung-Yun Shie, Ming-Hsui Tsai

**Affiliations:** ^1^Department of Otolaryngology, China Medical University Hospital, Taichung, Taiwan; ^2^Department of Audiology and Speech-Language Pathology, Asia University, Taichung, Taiwan; ^3^Department of Electrical Engineering, National Tsing Hua University, Hsinchu, Taiwan; ^4^Department of Sports Medicine, China Medical University, Taichung, Taiwan; ^5^Department of Biological Science and Technology, National Chiao-Tung University, Hsinchu, Taiwan

## Abstract

*Objective.* Autologous fat injection laryngoplasty is ineffective for some patients with iatrogenic vocal fold paralysis, and additional laryngeal framework surgery is often required. An acoustically measurable outcome predictor for lipoinjection laryngoplasty would assist phonosurgeons in formulating treatment strategies.* Methods.* Seventeen thyroid surgery patients with unilateral vocal fold paralysis participated in this study. All subjects underwent lipoinjection laryngoplasty to treat postsurgery vocal hoarseness. After treatment, patients were assigned to success and failure groups on the basis of voice improvement. Linear prediction analysis was used to construct a new voice quality indicator, the number of irregular peaks (NIrrP). It compared with the measures used in the Multi-Dimensional Voice Program (MDVP), such as jitter (frequency perturbation) and shimmer (perturbation of amplitude).* Results.* By comparing the [i] vowel produced by patients before the lipoinjection laryngoplasty (AUC = 0.98, 95% CI = 0.78–0.99), NIrrP was shown to be a more accurate predictor of long-term surgical outcomes than jitter (AUC = 0.73, 95% CI = 0.47–0.91) and shimmer (AUC = 0.63, 95% CI = 0.37–0.85), as identified by the receiver operating characteristic curve.* Conclusions.* NIrrP measured using the LP model could be a more accurate outcome predictor than the parameters used in the MDVP.

## 1. Introduction

Speech problems affect human communication. Degradation in voice quality can have a negative impact on a patient's daily life and in extreme cases can even lead to sociophobia [1]. This paper focuses on unilateral vocal fold paralysis (UVFP), which is a possible cause of dysphonia [2]. Iatrogenic UVFP caused by thyroid surgery can persist for 6 to 9 months after surgery. If the natural recovery process fails, patients may be required to undergo various types of phonosurgery, such as thyroplasty or injection laryngoplasty, to correct their voice impairment. However, few studies have reported on the outcome of lipoinjection laryngoplasty for iatrogenic UVFP after thyroid surgery.

Lipoinjection laryngoplasty is a conservative method for treating UVFP because autologous fat is a self-derived tissue that presents almost no tissue rejection concerns. It can also improve voice quality even in patients whose UVFP recovers naturally [3]. Moreover, the efficacy of lipoinjection laryngoplasty lasts for 12 months on average, reducing symptoms such as choking and glottal incompetence [4–6]. However, long-term outcomes are unpredictable because of reabsorption of the fat, with treatment failure rates of 30% after 2 years and 45% by 4 years [7]. Because of the unpredictable surgical outcome, repeated injections or laryngeal framework surgery such as thyroplasty are required. Preoperative prediction of a voice is therefore desirable both to improve patient selection for lipoinjection laryngoplasty and to ensure early intervention after recurrence of hoarseness [8, 9].

Existing tools and quality of life questionnaire for evaluation of vocal hoarseness include perceptional voice analysis such as the Voice Handicap Index (VHI), Consensus Auditory-Perceptual Evaluation of Voice (CAPE-V), acoustic analysis using the Multi-Dimensional Voice Program (MDVP) in a computer speech lab, and stroboscopic analysis [10]. The predictive power of jitter, shimmer, and the VHI for surgical outcomes of injection laryngoplasty has been reported in the literature, but no consensus has yet been reached on its effectiveness [10, 11]. Although some reports have claimed that lipoinjection laryngoplasty surgery reduces jitter and shimmer as measured using the MDVP for patients with UVFP, correlation with surgical outcomes has been inconsistent [10, 11]. Although autologous fat absorption can be detected early by using stroboscopy, it is difficult to evaluate in patients with a strong gag reflex [12].

MDVP has been found to be reliable and objective assessment software for voice quality in the patients with UVFP after injection laryngoplasty [13]. However, it has often failed in the biomedical signal analysis of highly degraded signals [14]. In this study, an acoustic inverse scattering technique called linear prediction (LP) was used to evaluate each patient's voice before and after lipoinjection. Huang et al. [15] found that LP could enhance the periodicity in noisy speech signals. LP, when used for voice synthesis purposes, could improve the perceptual voice quality and restore harmonic structure of the speech [15]. We therefore investigated an alternative method of clinically analyzing hoarse voice by identifying new acoustic parameters that can predict the outcome of lipoinjection laryngoplasty surgery in UVFP patients. The voice analysis method adopted in this study was LP, a mathematical technique that simultaneously estimates the GSW forms and the filtering effects provided by the vocal tract [16]. Because of its simultaneous source-filter estimation capability, LP has been widely used for digital speech communication purposes, such as speech synthesis and speech data compression [15]. In the current research, LP enabled us to focus on the GSW forms while ignoring the filtering effects of the pharynx and oral cavity. LP therefore is suitable for investigating the functions of the vocal fold without being influenced by confounding factors caused by vocal tract filtering. The aim of the study was to find a new acoustic predictor to improve patient selection and evaluation of postoperative outcomes.

## 2. Methods

### 2.1. Subjects

Between March 2012 and February 2014, UVFP patients underwent thyroid surgery in China Medical University Hospital and subsequently exhibited recurrent laryngeal nerve injury with hoarse voice involvement. Similarly as in the study by Jesus et al. this study assessed voice quality in patients with UVFP. Our sample size was 17 patients. All patients provided informed consent before lipoinjection laryngoplasty and the study was approved by the Institutional Review Board of the hospital. The diagnosis of UVFP was based on two criteria: laryngoscope assurance and the lack of laryngeal electromyography responses in the unilateral thyroarytenoid muscle. Following an observation period of 1 year, all patients received lipoinjection laryngoplasty for their hoarseness and choking problems. As noted, UVFP patients were divided into a success group and a failure group; the patient was assigned to the failure group if both the following criteria were met: the patient had recurrent nonrecoverable hoarseness, and the patient therefore underwent revision lipoinjection laryngoplasty or thyroplasty 6 months after the initial injection.

### 2.2. Autologous Fat Injection Laryngoplasty

**Figure 1:**
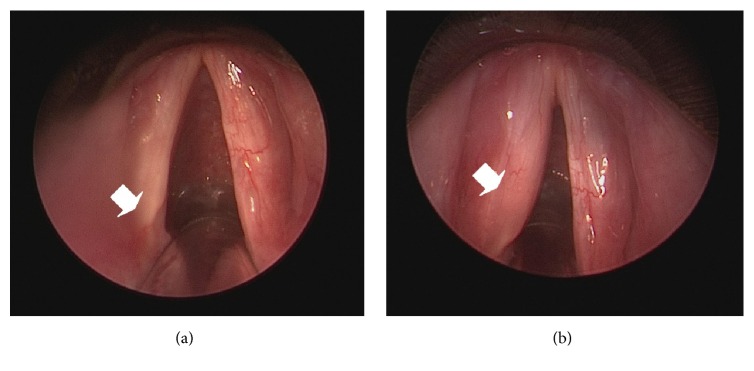
Paralyzed vocal fold before (a) and after (b) autologous fat for injection laryngoplasty.

Autologous fat for injection laryngoplasty was obtained from the periumbilical subcutaneous area. A 2 cm incision was made 0.5 cm beneath the umbilical area after local infiltration. Lidocaine hydrochloride (20 mL), dexamethasone (1 mL), 7% sodium bicarbonate (20 mL), and epinephrine (5 mg) were added to 500 mL of sodium chloride and mixed. Between 30 and 50 mL of the mixed solution was injected into the periumbilical subcutaneous area to elute fat for 5 minutes. Fat globules were then harvested using a 10 mL Storz injection syringe (Karl Storz, Tuttlingen, Germany). A total of 30–40 mL of subcutaneous adipose soft tissue was obtained and rinsed in 10 mL of regular insulin for 5 minutes after being washed in normal saline solution to remove blood clots. After soaking, the adipose tissue was loaded into a Storz Brünings-type laryngeal injector (Karl Storz, Tuttlingen, Germany) in preparation for injection. Patients were sedated using general anesthesia and a 5.5 or 6 mm oral endotracheal tube. A rigid suspension laryngoscope was used to expose the patient's vocal fold, and 1.5–2.0 mL of autologous fat was injected into the paralyzed side using an 18- or 19-gauge syringe. The injection point was at the posterior third of the membranous vocal fold, at the lateral aspect of the vocal process in the thyroarytenoid muscle. Injection in this point causes medicalization of the paralyzed vocal fold. In practice, 20–30% bulging of the paralyzed vocal fold across the midline was achieved after the injection (Figures 1(a) and 1(b)).

### 2.3. Voice Laboratory Measures

All of the patients underwent preoperative and postoperative acoustic recording and phonation studies. The perceptual evaluation of grade, roughness, breathiness, asthenia, and strain (using the GRBAS) and measurement of maximum phonation time (MPT) were performed by an otolaryngologist, Y-A T, before surgery and 6 months after it. Videostroboscopic examinations were also made, and they confirmed UVFP. The patients were asked to produce the vowels [a] and [i] at a stable pitch and loudness; the voice was then recorded and analyzed using the MDVP in a computerized speech laboratory system (CSL4500, Kay Elemetrics Corp, Lincoln Park, NJ, USA). The maximum phonation time was measured by the same phonosurgeon while patients produced a sustained [a] vowel. The midportion of the [a] and [i] vowel voice samples, which is considered a stable voice segment, was used for acoustic analysis. Fundamental frequency, jitter (frequency perturbation), shimmer (perturbation of amplitude), and harmonics-to-noise ratio (HNR) values were obtained using the MDVP [17].

All patients received laryngoscope and stroboscope examinations to survey the postoperation laryngeal gap, and the voices of the patients were recorded and analyzed using the MDVP both before the operation and at weekly intervals for 6 months after operation.

Based on their improvement in voice quality after the surgery, the patients were assigned to two groups. The lipoinjection treatment was considered a failure if the patient's voice was poorer within 6 months as determined using the MDVP or if an increased vocal slit was observed in stroboscopic analysis. Current clinical practice requires such patients to receive additional injections or permanent laryngeal framework surgery, such as medialization thyroplasty (silicon or Goretex). The lipoinjection treatment was considered to be a success if the patient's voice quality improved and stroboscopy showed that there was no slit during the maximal closure phase in the cycle of phonation.

### 2.4. Digital Signal Processing Methods

The digitally stored signals were analyzed offline by using computer programs written in MATLAB (Mathworks, Natick, MA, USA). The signal processing flow included preemphasis, windowing, LP, and feature extraction. The eventual goal was to examine the GSW form indicated by LP and to construct a useful predictor that might indicate the pathological status of the vocal fold. The signal processing methods are explained as follows.

The high-frequency components of the human voice have a roll-off tendency at a rate of 6 dB/octave when the sound waves radiate from the oral cavity. To compensate for this high-frequency attenuation, a filter was applied to the recorded signal to whiten the spectrum [18]:

(1) $$\begin{array}{c} s_{p}\left[\;n\;\right]=s\left[\;n\;\right]-0.95s\left[\;n-1\;\right], \end{array}$$

where *s*[*n*] denotes the raw signal at time *n* and *s*_*p*_[*n*] is the result after preemphasis. Spectrum whitening is a technique used to compensate for high-frequency attenuation of a signal within its own bandwidth in order to improve the resolution and appearance of voice data. This technique can prohibit excessive boosting of background noise that is not produced by a patient with UVFP.

In the field of speech processing, preemphasis is an essential step preceding LP analysis, because the LP error term (to be defined later) can be regarded as an effective representation of glottal waves only if the raw spectrum has no tendency to roll off. Equation (1) provides a 6 dB/octave boost that counteracts roll-off caused by radiative loss.

Because of the nonstationary property of the human voice, features of the voice signal were repeatedly extracted for each predetermined short period. The signal recorded within this period is referred to as a frame, and the rate at which features were extracted is called the frame rate. In this study, the length of a frame was set at 64 ms and the frame rate was the inverse of the length of the frame (15.625 frames/sec); in other words, frames did not overlap. In certain applications of spectral estimation, it is beneficial to multiply the frame with a windowing function to trade off resolution in the time domain and in the frequency domain. In this study, however, the frame was not further windowed by such a function; every sample maintained its original value after preemphasis.

The LP technique is employed to approximate every sample in a signal as a linear combination of previous samples. The approximation can be written as follows:

(2) $$\begin{array}{c} s_{p}\left[\;n\;\right]=\overset{N}{\underset{k=1}{\sum }}a_{k}s_{p}\left[\;n-k\;\right]+e\left[\;n\;\right], \end{array}$$

where {*a*_1_, *a*_2_,…, *a*_*n*_} are called the LP coefficients, *N* is the order of LP, and *e*[*n*] denotes the approximation error signal. When the LP coefficients are chosen to minimize the mean square of *e*[*n*], the spectrum of *e*[*n*] is maximally flat [19]. In practice, *e*[*n*] can be regarded as an estimation of the glottal source signal if *N* is sufficiently large and an optimal set of coefficients {*a*_1_, *a*_2_,…, *a*_*n*_} is found [20]. A vocal tract filter is simultaneously estimated, characterized by the acoustic transfer function *H*(*ω*):

(3) $$\begin{array}{c} H\left(\;\omega \;\right)=\frac{1}{1-\sum_{k=1}^{N}a_{k}e^{-ik\omega }}, \end{array}$$

where *ω* = 2*πf*/*f*_*s*_ is the digital frequency in rad/sample (where *f*_*s*_ denotes the sampling rate). Equations (3) and (2) can be rewritten as follows:

(4) $$\begin{array}{c} e\left[\;n\;\right]=s_{p}\left[\;n\;\right]-\overset{N}{\underset{k=1}{\sum }}a_{k}s_{p}\left[\;n-k\;\right]. \end{array}$$

This arrangement is interpreted as follows: *s*_*p*_[*n*] traverses the inverse filter of *H*(*ω*) so that any information concerning vocal tract filtering is removed. Therefore, the result *e*[*n*] can be regarded as a representation of the glottal source wave (GSW).

**Figure 2:**
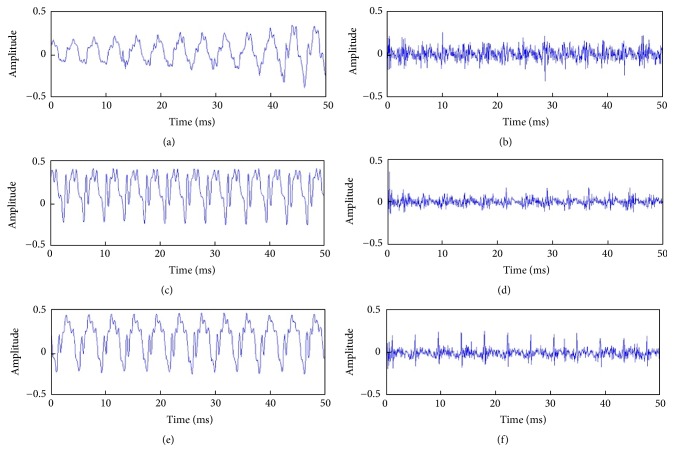
Voice signals processed by LP. Example of the raw recorded voice from one patient before, 1 month after, and 3 months after lipoinjection (a, c, e). The corresponding glottal source waves (GSWs) estimated by LP (b, d, f). By inspection, the postoperation glottal source waveforms (d, f) have more regular periodicity than the preoperation (b).

In the present study, *N* was fixed at 20, and the LP coefficients and the error (or excitation) signal *e*[*n*] were obtained iteratively through the Levinson-Durbin algorithm [21]. LP analysis was performed for every frame of the recorded signals.  Figure 2 shows typical results for GSWs, estimated using (4).

### 2.5. Statistical Analysis

The data were analyzed using the statistical analysis software SPSS 15. We used descriptive statistics to present the patients' demographic characteristics. Independent* t*-test was used to compare the success and failure groups, and paired* t*-test was used to determine the statistical difference between preoperative and postoperative GRBAS, MPT, and voice parameters for [a] and [i] vowels. The statistical significance of differences in gender distribution between the two groups was analyzed by Fisher's exact test. We plotted sensitivity against 1 − specificity between different voice parameters and new parameters for [a] and [i], to create receiver operating characteristic curves. The area under the curve (AUC) at a 95% confidence interval for each of the parameters was used to determine accuracy [22, 23].

## 3. Results

The patients ranged in age from 31 to 71 years (average = 54 y). The sex, age, and vocal problems of the patients are presented in Tables 1 and 2. The patients' fundamental frequency distribution was 140–230 Hz for the females and 70–190 Hz for the males. The failure group comprised 8 patients and the success group comprised 9 patients. There were 5 females and 4 males in the failure group, and 6 females and 2 males in success group. The two groups did not differ significantly from each other in terms of gender distribution (*p* = 0.62). After grouping, we analyzed voice parameters as follows.

### 3.1. A New Voice Quality Indicator

In the search for a new voice quality indicator, we first carefully inspected the GSWs, which are the voice signals originated from the vocal folds. As shown in Figure 2, when the lipoinjection surgery was successful, the GSWs indicated by LP became less noisy and prominent spikes emerged in the waveforms (as shown in Figure 2(f), compared with Figures 2(b) and 2(d)). This generated the following postulates: first, in a normal GSW, the prominent spikes should be of approximately equal height within a short frame; second, the prominent spikes should rise far above the noise floor, whereas the heights of other local maxima (or peaks) in the GSW are mostly near or below the noise floor.

**Figure 3:**
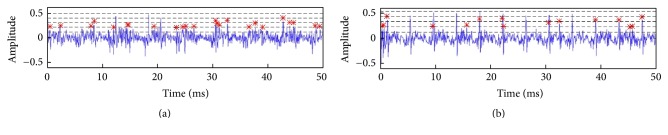
Peak counting in GSWs. A typical example from a patient in the failed group (a) and an example from a patient in the successful group (b) were shown. The lowest dashed line is the RMS value in the frame, and the highest line shows the maximum amplitude in the GSW of the frame. Peaks that fall within the 25% to 75% range between RMS and the maximum are marked with a red asterisk (*∗*).

Following these postulates, we constructed a new voice quality indicator, namely, the number of irregular peaks (NIrrP), by peak counting in each GSW frame. It represents the number of voice cycles whose peak amplitude was out of the preset range (below 25% and above 75%) in LP system. First, the root-mean-square (RMS) value of the GSW was treated as the noise floor (shown as the lowest dashed line in Figure 3). The maximum amplitude was then identified and the amplitude range of the GSW was partitioned into four equal regions between the RMS value and the maximum (shown as the highest dashed line in Figure 3). Finally, peaks whose height fell within the 25–75% range between RMS and maximum were counted (marked with red asterisks in Figure 3). The NIrrP is defined as the average number of these peaks in each 64 ms frame in the GSW. In a failed case, the GSWs should appear noisy and glottal spikes should be obscured. Therefore, we expected the NIrrP to be higher in the failure group (see Figure 3(a)) than in the success group. Comparing with failure group, significant differences in NIrrP and MDVP of [a] and [i] vowels in postoperation were found in success group (*p* < 0.05; Table 3).

### 3.2. Comparing NIrrP against MDVP Parameters

The accuracy of the NIrrP in predicting surgical outcomes was compared with that of the parameters calculated by the MDVP, such as jitter, shimmer, and HNR. The aim was to ascertain whether NIrrP and other parameters can be used to predict the long-term outcome of lipoinjection surgery. We first made a preoperative comparison between the success and failure groups across each of the parameters. The MDVP parameters and NIrrP for the failure and success groups are shown in Table 3. Comparing the voice samples recorded before and after surgical operation, no other statistically significant differences were observed, except for jitter of vowels [a] and [i] and NIrrP of vowels [i]. The failure and success groups did not differ from each other for the jitter and shimmer values measured by MDVP before surgery. However, after surgery the groups differed significantly for GRBAS, and the jitter and shimmer values from vowels [a] and [i]. A statistically significant difference was found for the new voice parameters of the NIrrP tested in preoperative testing on [i] vowels (success group = 31.48 ± 7.62; failure group = 51.30 ± 8.03; *p* = 0.004), whereas no statistically significant differences were found in preoperative testing on the [a] vowel (success group = 37.82 ± 14.77; failure group = 43.71 ± 12.35; *p* = 0.39). These results identified some MDVP parameters that may help to differentiate better and worse voice qualities from each other and thus indicate the success of a surgical operation. Furthermore, the results suggested that a new voice parameter, NirrP, derived from patients' [a] and [i] vowels, may also provide a sensitive indicator of voice quality.

**Figure 4:**
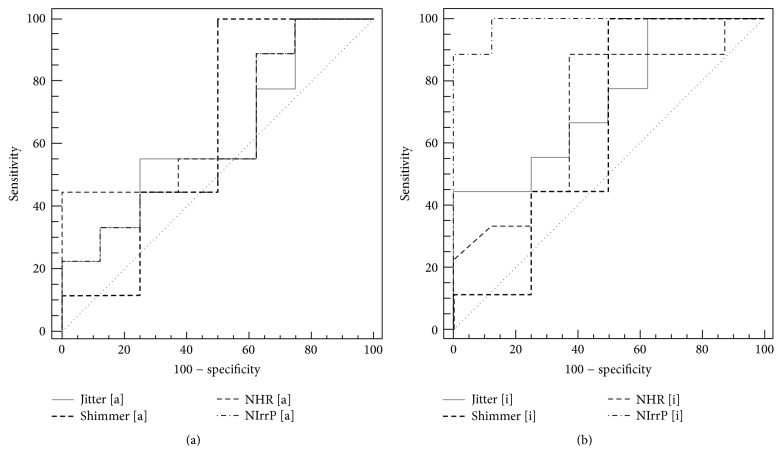
The predictive power of the parameters by AUC under ROC. The NIrrP could not be a significantly prognostic marker for outcome of lipoinjection laryngoplasty by the vowel [a] (a). The NIrrP could be a significantly prognostic marker for outcome of lipoinjection laryngoplasty by the vowel [i] (b).

We further quantified the predictive power of the parameters by AUC under receiver operating characteristic curve (ROC). The results showed that the new voice parameter [i] of NIrrP is the most favorable among all parameters for discriminating between successful and unsuccessful outcomes before autologous fat injection laryngoplasty (Figures 4(a) and 4(b)). The ROC for the new voice parameter [i] (AUC = 0.98, 95% CI = 0.78–0.99) was significantly higher than the voice parameter [i], jitter (AUC = 0.73, 95% CI = 0.47–0.91), shimmer (AUC = 0.63, 95% CI = 0.37–0.85), and HNR (AUC = 0.70, 95% CI = 0.43–0.89). However, no significant difference (*p* > 0.05) was observed between the NIrrP for [a] (AUC = 0.71, 95% CI = 0.41–0.83) and the MDVP voice parameters for [a] (Table 4).

## 4. Discussion

Permanent recurrent laryngeal nerve injury may occur after surgery for thyroid cancer or thyroid nodular goiter and may require laryngeal surgery [24]. Thyroplasty is typically the first choice to treat permanent UVFP and stable results have been presented in the literature [25]. However, foreign body reaction and migration of implants may appear in long-term follow-up. In addition, the neck will have an additional wound and fibrotic scar which may also cause implant migration. Furthermore, patients undergoing thyroplasty still have residual glottis insufficiency and salvage injection laryngoplasty is still required [26, 27]. Therefore, autologous fat injection laryngoplasty offers an alternative treatment strategy. Controversy remains over the choice of injection laryngoplasty or thyroplasty for UVFP patients following thyroid surgery. However, no superior methods for the prognosis of injection laryngoplasty have been reported in the literature. GRBAS, CAPE-V, jitter, shimmer, and HNR have been used to compare patients' voice quality before and after various types of laryngeal surgery [28]. In particular, voice grade has been reported to be a predictor for the surgical outcome of thyroplasty [29]. However, none of these parameters are able to predict the surgical outcome of injection laryngoplasty. Therefore, a reliable outcome predictor is needed to allow phonosurgeons to improve surgical decision making.

MPT and GRBAS are commonly used for evaluation of patient's voice in clinic and are subjective voice evaluation for vocal fold paralysis [30]. In the results of previous study, a significantly shorter MPT was found in the patients with vocal fold paralysis to compare with normal subjects and was an appropriate predictor of outcome after thyroplasty [31]. The results reported by Morsomme et al. [32] also indicated that reduced GRBAS reflected the success of UVFP treatment. Increased MPT and a decrease in GRBAS would be expected after a successful lipoinjection laryngoplasty. However, in the present study only an increase in MPT was found in both the success and failure groups after lipoinjection laryngoplasty, while no changes were found in GRBAS. The reason may be that lipoinjection laryngoplasty improves the glottic closure efficiency, and MPT was useful to assess the improvements [33]. GRBAS is emphasized in the perceptual assessment of voice quality [32]. Acoustic parameters such as fundamental frequency, jitter, shimmer, and HNR provide possibility for an objective evaluation of voice quality, which complements perceptual voice evaluation. According to Zhang et al. [34], the pathological voice of UVFP patients had higher jitter and shimmer values compared to normal voices. In the present study, the receiver operating characteristic analysis was used to assess the jitter and shimmer characteristics in the patients' [a] and [i] vowels. The acceptable discrimination of sensitivity and specificity of these two voice parameters in distinguishing the success group of patients with UVFP from failure group was found before autologous fat injection laryngoplasty. These findings were the same as previous studies. In our study, NIrrP of the patients' [i] vowel excellently differentiated the success and failure groups after lipoinjection laryngoplasty from each other. NIrrP in our model behaved more precisely than jitter and shimmer in detection of cycle-to-cycle variations in period length and amplitude of the voice signal. Thus, it could be a predictor of voice quality, indicating stability of vocal fold vibration, to be used before and after phonosurgery.

Like GRBAS, CAPE-V is measured subjectively; the jitter, shimmer, and HNR are obtained objectively by using the MDVP. However, these factors are affected by the vocal tract. This might explain their poor performance as surgical outcome predictors. By contrast, LP measures the source of the voice produced by the vocal folds and removes the filtering effects of the vocal tract [31]. In this research, the NIrrP test on the [i] vowel showed a significant ability to differentiate between the success and failure groups both before operation and after operation. The AUC of the NIrrP test (>0.9) was higher than that of jitter and shimmer [23]. The NIrrP had greater predictive power than jitter and shimmer even when tested after 6 months in [i] production, but not in [a] production. These results could be explained by the higher vocal tension of the thyroarytenoid muscle and cricothyroid muscle in production of the [i] vowel [35–37]. This increased vocal tension allows more effective vocal fold closure for the [i] vowel than the [a] vowel. Testing on [i] also showed less jitter and shimmer than that on [a] and reduced interference of noise from irregular movements during vocal fold vibration. Therefore, voice analysis on the [i] vowel provided more significant changes in predicting surgical outcomes. A recent study conducted tests on different vowels and attributed dysphonia to the different supraglottal changes during [a] and [i] phonation, which, in turn, affect vocal tract resonances [32]. A dynamic magnetic resonance imaging study demonstrated the different movement of the vocal folds for [a] and [i] vowels and revealed a lower larynx position in [a] vowel production than in [i] vowel production [33]. However, differences in testing [a] and [i] persist even in the LP model, in which voicing comes directly from the vocal fold and is not affected by the actions of the vocal tract, such as the pharynx, oral cavity, and tongue.

Our focus was on identifying outcome predictors for lipoinjection laryngoplasty using the LP model. We found that jitter and shimmer were weaker predictive factors than the NIrrP in [i] before the operation and 6 months after the operation. Further research is needed to explore these results in more detail. We also suggest extending the research to the production of different vowels, such as /u/, /e/, and /o/. A limitation of the study is the lack of any measure of thyroarytenoid muscle and cricothyroid muscle tension. We postulated that the [i] vowel requires higher thyroarytenoid and cricothyroid muscle tension and that this might directly produce different NIrrP results in the LP model. Our participant group was also relatively small; to establish more robust results, replication with larger groups of participants is recommended.

The NIrrP can be used as a reliable preoperative predictor of surgical outcomes. The NIrrP in [i] vowel testing in the LP model for lipoinjection laryngoplasty produced accurate outcome predictions both before surgery and 6 months afterward. This approach can assist phonosurgeons in making more precise diagnoses and improve patient selection for this type of surgery. The predictive power of the NIrrP in [i] vowel testing for lipoinjection laryngoplasty has not been previously reported, and this technique should be applied more widely in clinical practice.

## Figures and Tables

**Table 1:** Basic data regarding all patients before operation.

Number	Sex	Age	Lesion site	GRBAS	MPT
1	Female	59	Rt UVFP	22331	4
2	Female	67	Rt UVFP	22111	3
3	Male	43	Rt UVFP	22222	5
4	Male	55	Rt UVFP	22231	4
5	Male	68	Lt UVFP	22111	4
6	Male	31	Lt UVFP	23322	5
7	Male	71	Rt UVFP	23112	4
8	Male	61	Lt UVFP	22222	4
9	Female	55	Rt UVFP	22221	8
10	Female	46	Lt UVFP	22311	8
11	Female	59	Lt UVFP	22311	5
12	Female	49	Rt UVFP	22331	5
13	Female	48	Rt UVFP	22222	5
14	Female	42	Lt UVFP	23322	4
15	Female	59	Lt UVFP	22222	5
16	Female	49	Lt UVFP	22322	5
17	Female	48	Rt UVFP	22231	5

Lt, left side; Rt, right side; GRBAS, grade, roughness, breathiness, asthenia, and strain scale; MPT, maximum phonation time; UVFP, unilateral vocal fold paralysis.

**Table 2:** Patient characteristics.

	Overall (*n* = 17)	
	Number (percent)	Mean (SD)
Average age		53.53 (10.47)
Sex		
Male	6 (35.29%)	
Female	11 (64.71%)	
Site of lesion		
Rt	9 (52.94%)	
Lt	8 (47.06%)	
Etiology		
Iatrogenic after thyroid surgery	17 (100%)	
GRBAS		
Grade		2.00 (0.00)
Roughness		2.18 (0.39)
Breathiness		2.24 (0.75)
Asthenia		1.94 (0.75)
Strain		1.47 (0.51)
MPT		4.88 (1.32)

Lt, left side; Rt, right side; GRBAS, grade, roughness, breathiness, asthenia, and strain scale; MPT, maximum phonation time.

**Table 3:** The results of success and failure groups before and after operation.

	Overall (*n* = 17)		Success group (*n* = 9)		Failure group (*n* = 8)	
	Pre	Post	Pre	Post	Pre	Post
GRBAS	2.02 (0.51)	1.60 (0.72)	2.02 (0.41)	1.02 (0.29)^+^	2.02 (0.62)	2.23 (0.45)^*∗*^
MPT	5.04 (1.91)	16.06 (8.27)	5.36 (1.92)	15.72 (8.14)^+^	4.67 (1.95)	16.45 (8.96)^+^
Voice [a]						
Jitter	4.57 (4.09)	1.59 (1.30)^+^	3.42 (2.45)	0.81 (0.42)^+^	5.87 (5.27)	2.48 (1.41)^*∗*^
Shimmer	0.85 (0.76)	0.59 (0.37)	0.56 (0.31)	0.34 (0.17)	1.18 (1.01)	0.88 (0.34)^*∗*^
NHR	0.21 (0,15)	0.17 (0.11)	0.16 (0.08)	0.12 (0.03)	0.26 (0.19)	0.23 (0.14)
NIrrP	40.59 (13.60)	34.70 (20.51)	37.82 (14.77)	35.17 (19.29)	43.71 (12.35)	34.16 (13.15)^*∗*^
Voice [i]						
Jitter	4.12 (3.04)	1.96 (1.74)^+^	2.86 (1.82)	0.68 (0.32)^+^	5.53 (3.61)	3.41 (1.52)^*∗*+^
Shimmer	0.85 (0.76)	0.70 (0.47)	0.56 (0.30)	0.43 (0.31)	1.18 (1.01)	1.02 (0.44)^*∗*^
NHR	0.16 (0.13)	0.21 (0.13)	0.12 (0.05)	0.14 (0.01)	0.21 (0.17)	0.30 (0.14)^*∗*^
NIrrP	43.16 (11.91)	32.32 (16.23)^+^	31.48 (7.62)	31.34 (15.62)	51.30 (8.03)	33.43 (17.91)^*∗*+^

^*∗*^ *p* < 0.05, success versus failure group; ^+^ *p* < 0.05, preoperation versus postoperation.

GRBAS, grade, roughness, breathiness, asthenia, and strain scale; MPT, maximum phonation time; NHR, noise to harmonic ratios.

**Table 4:** The analysis of ROC curve between different voice parameters.

	AUC	SE	95% CI
GRBAS	0.52	0.15	0.27–0.76
MPT	0.65	0.15	0.38–0.86
Voice [a]			
Jitter	0.62	0.14	0.36–0.84
Shimmer	0.63	0.15	0.37–0.85
NHR	0.66	0.14	0.40–0.87
NIrrP	0.71	0.14	0.41–0.83
Voice [i]			
Jitter	0.73	0.12	0.47–0.91
Shimmer	0.63	0.15	0.37–0.85
NHR	0.70	0.13	0.43–0.89
NIrrP	0.98	0.01	0.78–0.99

AUC, area under curve; SE, standard error; GRBAS, grade, roughness, breathiness, asthenia, and strain scale; MPT, maximum phonation time; NHR, noise to harmonic ratios; NIrrP, number of irregular peaks.
